# Neighbors’ use of water and sanitation facilities can affect children’s health: a cohort study in Mozambique using a spatial approach

**DOI:** 10.1186/s12889-022-13373-9

**Published:** 2022-05-16

**Authors:** Berta Grau-Pujol, Jorge Cano, Helena Marti-Soler, Aina Casellas, Emanuele Giorgi, Ariel Nhacolo, Francisco Saute, Ricard Giné, Llorenç Quintó, Charfudin Sacoor, Jose Muñoz

**Affiliations:** 1 Barcelona Institute for Global Health, Hospital Clínic - Universitat de Barcelona, C/Rosselló 132 4°1ª, 08036 Barcelona, Spain; 2 Centro de Investigação em Saúde de Manhiça (CISM), Maputo, Mozambique; 3 Mundo Sano Foundation, Buenos Aires, Argentina; 4 Expanded Special Project for Elimination of NTDs, World Health Organization Regional Office for Africa, Brazzaville, Congo; 5 Departament de Fonaments Clínics, Facultat de Medicina, Universitat de Barcelona (UB), Casanova 143, 08036 Barcelona, Spain; 6 Lancaster Medical School, Faculty of Health and Medicine, Lancaster University, Bailrigg, Lancaster, LA1 4YW UK; 7 Stockholm International Water Institute, Stockholm, Sweden

**Keywords:** Water, Sanitation, Wash, Herd protection, Community coverage, Morbidity, Wasting, Africa, Spatial, Health care

## Abstract

**Background:**

Impact evaluation of most water, sanitation and hygiene (WASH) interventions in health are user-centered. However, recent research discussed WASH herd protection – community WASH coverage could protect neighboring households. We evaluated the effect of water and sanitation used in the household and by household neighbors in children’s morbidity and mortality using recorded health data.

**Methods:**

We conducted a retrospective cohort including 61,333 children from a district in Mozambique during 2012–2015. We obtained water and sanitation household data and morbidity data from Manhiça Health Research Centre surveillance system. To evaluate herd protection, we estimated the density of household neighbors with improved facilities using a Kernel Density Estimator. We fitted negative binomial adjusted regression models to assess the minimum children-based incidence rates for every morbidity indicator, and Cox regression models for mortality.

**Results:**

Household use of unimproved water and sanitation displayed a higher rate of outpatient visit, diarrhea, malaria, and anemia. Households with unimproved water and sanitation surrounded by neighbors with improved water and sanitation high coverage were associated with a lower rate of outpatient visit, malaria, anemia, and malnutrition.

**Conclusion:**

Household and neighbors’ access to improve water and sanitation can affect children’s health. Accounting for household WASH and herd protection in interventions’ evaluation could foster stakeholders’ investment and improve WASH related diseases control.

**Graphical Abstract:**

Distribution of main water and sanitation facilities used during study period.
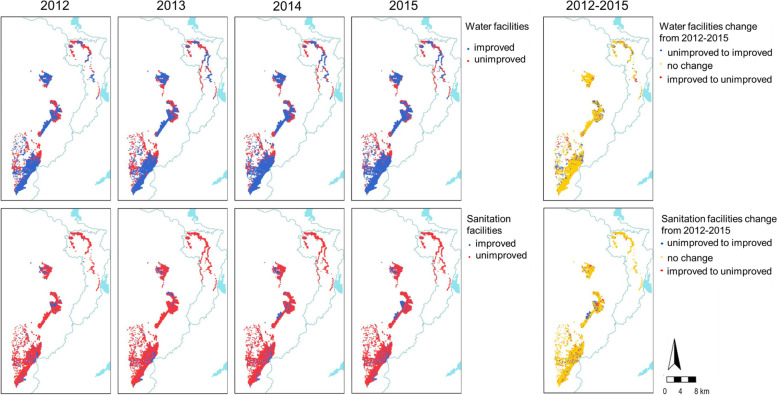

**Supplementary Information:**

The online version contains supplementary material available at 10.1186/s12889-022-13373-9.

## Background

Safe drinking-water supply, basic sanitation, and hygiene (WASH) are essential for good health. Poor access to these services favors fecal-oral transmission of infectious diseases and vector borne diseases, among others [1–3].

Globally, 70% of the population was estimated to have access to safely managed drinking water and 40% used safely managed sanitation services in 2017; in Sub-Saharan Africa, this corresponds to a 27 and 18% of the population respectively [4]. The international community considers the access to safe and protected water and improved sanitation services a target goal in the Sustainable Development Goal (SDG) 6 of the 2030 Agenda [5].

The connection between WASH and human health have been largely studied. For instance, treated piped water may reduce diarrhea risk up to 75% compared to the use of unimproved drinking water [6]; the risk of anemia has been found to be lower in households with toilet available [7]; and absence of toilet has been associated with a higher risk of malnutrition [8]. Nonetheless, some studies have not been able to find association because of limitations on the study design: most research utilizes self-reported health data, their design only focuses on household WASH exposure or, in the case of cluster randomized trials, they are limited by low adherence to the WASH intervention [9, 10]. Recent studies discussed that access to improved WASH can also protect the community: improved water and sanitation facilities’ community coverage could contribute to protect neighboring households of pathogen infection. This phenomenon is called herd protection and it is poorly studied in WASH [11].

We conducted a retrospective cohort study in southern Mozambique to evaluate the linkages between the quality of water and sanitation facilities used in the household and by household neighbors with health care-based children morbidity and mortality recorded data during 2012–2015. In particular, we studied the association with outpatient visit, hospital admission, diarrhea, malaria, anemia, malnutrition, dehydration and mortality.

## Methods

### Study area and study population

Manhiça district is a peri-urban area in Southern Mozambique located 80 km from the capital. The elevation of the area ranges from 30 m to 130 m. Climate there is subtropical with a warm and rainy season (November to April) and a cool and dry season (June to October). The average annual temperatures oscillate from 22 °C to 24 °C and the average annual precipitation from 600 mm to 1000 mm [12]. National coverage of improved drinking water and improved sanitation were 71.9 and 38.5% respectively in 2017 [13].

Since 1996, the Centro de Investigação em Saúde de Manhiça (CISM) conducts a demographic surveillance system (DSS) for vital events and migrations in Manhiça District. The DSS also records household parameters, household geoposition and living conditions. In addition, for inhabitants under 15 years old, DSS collects routine morbidity data and in- and outpatient visits to the District hospital and five health centers within the DSS area. DSS residents have a unique identifier (PermID) which enables to update their demographic status (i.e. population movements, mortality, etc.) and register their path through the health system [12].

In 2012, DSS covered a region with nearly 99,000 inhabitants, 56% were female and 41% were < 15 years of age. Villages encompass a loose conglomeration of compounds separated by yards and cropping land. The main occupations are farming, petty trading and working on a sugar cane estate [12]. Diarrhea accounted for 20% of paediatric hospital admissions in 2013 [12]; malaria is endemic and severe malnutrition is a common cause of outpatient visit [14, 15]. Further details of CISM DSS are described elsewhere [12].

### Study design

We conducted a retrospective cohort study including all children under age 15 living in DSS area during 2012–2015. Children were included in the study first day (January 1st, 2012), the birth date or the immigration date (when they started living in the DSS area), whatever occurred later. They were followed-up until they moved out from the DSS area, turned age 15 or, if neither occurred, until the study last day on December 31st' 2015.

We obtained water and sanitation household data from the DSS [12]. The study variables were: i) main water facility used in the household, and ii) main sanitation facility used in the household. The variables were dichotomized as “improved” and “unimproved” as defined by the WHO/UNICEF Joint Monitoring Program. Briefly, an “improved” drinking-water source is one that “by the nature of its construction or through active intervention, is protected from outside contamination, particularly fecal matter”. An “improved” sanitation facility is one that “safely separates excreta and wastewater from human contact either by safe containment and disposal in situ or by safe transport and treatment off-site” [16]. Thus, we considered improved facilities toilet connected to septic tank, improved latrine, piped water inside the household, piped water outside the household, fountain and pumped well. Unimproved latrine, open defecation, well without a pump and surface water were considered unimproved. Data on hygiene habits and hand washing at household level was not collected, therefore we could only include water and sanitation facilities used in our analysis [17].

We obtained morbidity and mortality data through the DSS morbidity surveillance system for outpatient and hospital admission at the Manhiça District Hospital and health centers [12]. We studied the following morbidity indicators: i) hospital or health center outpatient visit, ii) hospital admission, iii) diarrhea diagnosis (> three stools per day), iv) clinical malaria diagnosis, v) anemia (hematocrit levels < 33%), vii) malnutrition (low weight-for-height), viii) dehydration (loss skin elasticity, reduced or absent urine flow, normal to slightly sunken eyes and sunken fontanelle in infants), and ix) mortality.

A socioeconomic wealth index based on household characteristics and assets possession from DSS data to attribute a household socioeconomic status (SES) was constructed [18]. We performed a multiple correspondence analysis (MCA) to determine the weights of every characteristic or asset [13]. We included 18 variables: house construction type, house construction material, kitchen location, kitchen coverage, main cooking fuel, electricity supply, certain assets possession (telephone, radio, video or DVD, fridge, car or tractor, television, computer and stove), farming activity and literacy, education and occupation of the head of the household. We excluded water and sanitation variables to avoid over adjustment. Further details on how the SES was constructed is provided in an additional file (Additional file 1).

### Data analysis

Participant population (age, sex, neighborhood of residence, water and sanitation facilities used) was described using mean and standard deviation, and absolute and relative frequency for continuous and discrete variables, respectively. A non-parametric trend test was used to assess variables variation along study period.

We estimated the incidence rates for every morbidity indicator. We calculated time at risk as the number of children years at risk since study inclusion until the end of follow-up. After each episode, we applied a lag period for each outcome, except mortality. Lag periods were discussed and decided by a clinical experts committee, they were the following: outpatient visit 1 day, hospital admission 15 days, diarrhea 15 days, dehydration 15 days, malnutrition 15 days, anemia 30 days and malaria 28 days. During lag periods, children did not contribute to time at risk or cases. We expressed incidences as episodes per 100 CYAR (Children Years at Risk). Due to the overdispersion of the data, we fitted negative binomial regression models. We calculated minimum children-based incidence rates (MCBIR) for every morbidity indicator referring cases to population denominators establishing time at risk inferred from DSS information. We estimated models with random intercept to consider repeated measures. For mortality, we fitted a Cox regression model. The models were selected using backward procedure. We adjusted our estimations for age, sex, SES, season and distance to the closest health center (Euclidean distance). Our references were piped water inside the household and toilet connected to a septic tank.

To evaluate herd protection, we studied the association of the density of neighboring households with improved facilities considering household facility with the morbidity events. We estimated neighboring density using a Kernel Density Estimator, a non-parametric way to estimate the probability intensity function of a random variable. We assumed the random variable (water and sanitation indicators) to be a stationary (homogeneous) Poisson process. The optimal bandwidth for each intensity function was estimated so that it would be the one that minimizes the mean square error, as described by Diggle [19]. The analysis was conducted using the *spatstat* R package intended for the analysis of spatial point patterns. The results of applying the fitted intensity function to the water and sanitation indicators were fed into a spatial grid of 100 × 100 m resolution. We divided the estimated density for both improved water and improved sanitation facilities in four density quartiles to classify improved facilities coverage in household neighbors (from higher to lower): i) high coverage, ii) medium-high coverage iii) medium-low coverage and iv) low coverage (Additional file 2). For water, we created a herd protection variable of eight categories, we combined household improved or unimproved facilities with improved facilities coverage in household neighbors (high coverage, medium-high coverage, medium-low coverage and low coverage), e.g. household water improved facilities surrounded with high water coverage. We created the same herd protection variable for sanitation. We fitted a negative binomial regression model for all the morbidity outcomes. For the mortality, we implemented a Cox regression model. Models were constructed with the same confounders mentioned above using backward procedure. For herd protection water and sanitation variables, we used the reference categories “household improved facilities surrounded with high coverage” and “household unimproved facilities surrounded by low coverage”.

We performed statistical analysis and data management and visualization using STATA 16 (StataCorp., TX, USA) and R Statistical Software Version 3.5.3 [20].

## Results

### Water and sanitation characteristics in the study population

**Fig. 1:**
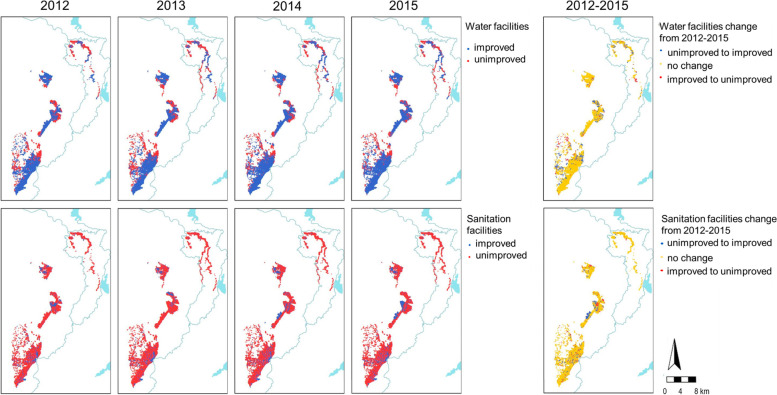
Distribution of main water and sanitation facilities used per study participants household during 2012–2015. Base layer map obtained in https://data.humdata.org/dataset/mozambique-administrative-levels-0-3, map edited using R Statistical Software Version 3.5.3

Between 2012 and 2015, we included 61,333 children under 15 years old from Manhiça District in the cohort. At baseline, half of them (50.1%) were males and 22.1% were between 0 and 2 years old. Then, 77.6% of children used an improved water facility and 21.1% of them used an improved sanitation facility at home. In 2015, the proportion of children leaving in a household with improved water facility slightly improved to 85.3%, but only 23.3% had improved sanitation facilities to date (Fig. 1 and Table 1).

**Table 1:** Description of study population during years 2012–2015

	2012	2013	2014	2015	*p*-value*
	n (%)	n (%)	n (%)	n (%)	
**Sex**					0.537
Male	20,388 (50.1)	23,731 (50.3)	24,000 (50.6)	23,434 (50.5)	
Female	20,318 (49.9)	23,405 (49.7)	23,477 (49.4)	22,987 (49.5)	
**Total**	**40,706 (100.0)**	**47,136 (100.0)**	**47,477 (100.0)**	**46,421 (100.0)**	
**Age**					< 0.001
< 2	8987 (22.1)	8659 (18.4)	8309 (17.5)	6995 (15.1)	
2–4	8034 (19.7)	9612 (20.4)	9564 (20.1)	9557 (20.6)	
5–9	12,693 (31.2)	15,424 (32.7)	15,756 (33.2)	15,851 (34.1)	
10–14	10,992 (27.0)	13,441 (28.5)	13,848 (29.2)	14,018 (30.2)	
**Total**	**40,706 (100.0)**	**47,136 (100.0)**	**47,477 (100.0)**	**46,421 (100.0)**	
**Location**					< 0.001
Maciana	8726 (21.4)	9662 (20.5)	9808 (20.7)	9562 (20.6)	
Cambeve	3763 (9.2)	4332 (9.2)	4433 (9.3)	4303 (9.3)	
Manhiça-sede	10,563 (25.9)	12,319 (26.1)	12,322 (26.0)	12,265 (26.4)	
Manchiana	2863 (7.0)	2907 (6.2)	2913 (6.1)	2864 (6.2)	
Palmeira	8042 (19.8)	10,171 (21.6)	10,182 (21.4)	9874 (21.3)	
Taninga	2862 (7.0)	3289 (7.0)	3340 (7.0)	3278 (7.1)	
Ilha Josina Machel	3887 (9.5)	4456 (9.5)	4479 (9.4)	4275 (9.2)	
**Total**	**40,706 (100.0)**	**47,136 (100.0)**	**47,477 (100.0)**	**46,421 (100.0)**	
**Water source conditions**					< 0.001
*Improved*	31,580 (77.6)	36,977 (78.4)	39,263 (82.7)	39,588 (85.3)	
*Unimproved*	9126 (22.4)	10,159 (21.6)	8214 (17.3)	6833 (14.7)	
**Total**	**40,706 (100.0)**	**47,136 (100.0)**	**47,477 (100.0)**	**46,421 (100.0)**	
Piped water inside	4696 (11.5)	6658 (14.1)	7238 (15.2)	9157 (19.7)	< 0.001
Piped water outside	12,469 (30.6)	16,255 (34.5)	18,412 (38.8)	17,764 (38.3)	
Fountain	10,215 (25.1)	7944 (16.9)	6246 (13.2)	5599 (12.1)	
Pumped well	4200 (10.3)	6120 (13.0)	7367 (15.5)	7068 (15.2)	
Surface water	39 (0.1)	18 (0.0)	92 (0.2)	36 (0.1)	
Well without a pump	9087 (22.3)	10,141 (21.5)	8122 (17.1)	6797 (14.6)	
**Total**	**40,706 (100.0)**	**47,136 (100.0)**	**47,477 (100.0)**	**46,421 (100.0)**	
**Sanitation conditions**					< 0.001
*Improved*	8584 (21.1)	8817 (18.7)	10,685 (22.5)	10,828 (23.3)	
*Unimproved*	32,122 (78.9)	38,319 (81.3)	36,792 (77.5)	35,593 (76.7)	
**Total**	**40,706 (100.0)**	**47,136 (100.0)**	**47,477 (100.0)**	**46,421 (100.0)**	
Toilet with septic tank	1797 (4.4)	2620 (5.6)	2034 (4.3)	1860 (4.0)	< 0.001
Improved latrine	6787 (16.7)	6197 (13.1)	8651 (18.2)	8968 (19.3)	
Unimproved latrine	30,840 (75.8)	37,065 (78.6)	35,449 (74.7)	34,380 (74.1)	
Without latrine	1282 (3.1)	1254 (2.7)	1343 (2.8)	1213 (2.6)	
**Total**	**40,706 (100.0)**	**47,136 (100.0)**	**47,477 (100.0)**	**46,421 (100.0)**	

* Non-parametric test for trend for year

### Association between water and sanitation household facilities with morbidity indicators

#### Association between household water facility used with morbidity indicators

**Fig. 2:**
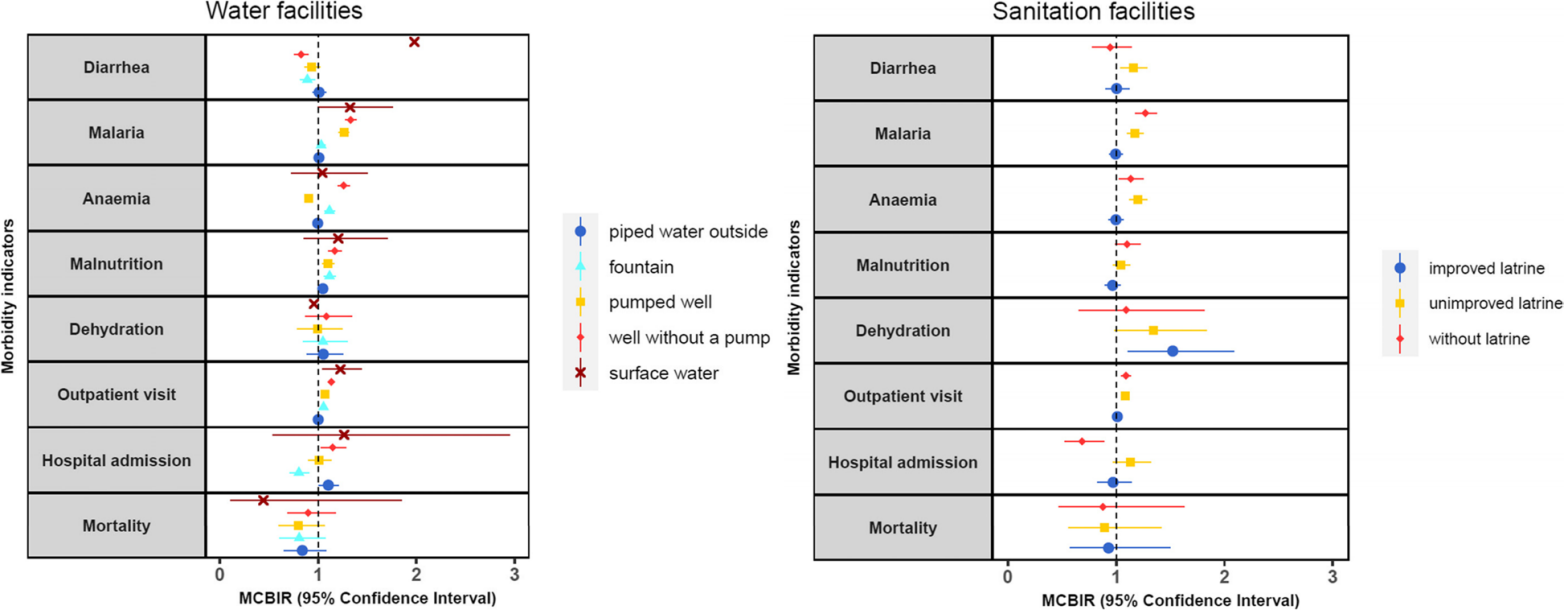
Minimum children-based incidence rates (MCBIR) for diarrhea, malaria, anemia, malnutrition, dehydration, outpatient visits, hospital admission and mortality per main water source and sanitation facilities household use during 2012–2015 in Manhiça district adjusted for age, sex, SES, season and distance to health center. The reference categories were the use of piped water inside the household and toilet connected to a septic tank

Households using unimproved water facilities (well without a pump or surface water) showed fair evidence of a higher minimum children-based incidence rate (MCBIR) for diarrhea, malaria, anemia, malnutrition, outpatient visit and hospital admission in children compared to those with piped water inside the household, after controlling for age, sex, SES, season, and distance to health center. Specifically, diarrhea rate was doubled with surface water usage (MCBIR 1.98, 95%CI 1.16–3.38, *P* <  0.001). In addition, households using surface water also had a higher outpatient visit rate (MCBIR 1.23, 95%CI 1.05–1.44, *P* <  0.001). Well without a pump use was associated with greater risk for malaria, anemia, malnutrition, outpatient visit and hospital admission, but a lower risk for diarrhea (MCBIR 0.83, 95%CI 0.76–0,90, *P* <  0.001). The rate of anemia (MCBIR 1.12, 95%CI 1.07–1.17, *p* <  0.001) and malnutrition (MCBIR 1.12, 95%CI 1.06–1.18, *P* <  0.001) was also moderately higher for those household accessing fountain water, but it was moderately lower for diarrhea (MCBIR 0.89, 95%CI 0.82–0,97, *P* <  0.001) and hospital admission (MCBIR 0.81, 95%CI 0.72–0,91, *P* <  0.001). Dehydration and mortality were not associated with any type of water facilities after adjusting for confounders (Fig. 2 and Additional file 3).

#### Association between household sanitation facility used with morbidity indicators

Children living in a household using unimproved sanitation facilities (unimproved latrine or not having a latrine at home) were associated with a larger minimum children-based incidence rate for diarrhea, malaria, anemia and outpatient visit compared to toilet connected to septic tank use, after controlling for age, sex, SES, season and distance to health center. In particular, not having a latrine at home was associated with a higher rate for malaria (MCBIR 1.27, 95%CI 1.17–1.38, *P* <  0.001), anemia (MCBIR 1.14, 95%CI 1.03–1.25, *P* <  0.001) and outpatient visit (MCBIR 1.09, 95%CI 1.05–1.38, *P* <  0.001). Moreover, households with unimproved latrine had a greater rate of diarrhea (MCBIR 1.16, 95%CI 1.04–1.29, *P* <  0.001), malaria (MCBIR 1.18, 95%CI 1.11–1.25, *P* <  0.001), anemia (MCBIR 1.20, 95%CI 1.13–1.29, *P* < 0.001) and outpatient visit (MCBIR 1.08, 95%CI 1.05–1.11, *P* < 0.001). Households using an improved latrine also exhibit a higher dehydration rate (MCBIR 1.52, 95%CI 1.11–2.09*, P* = 0.030). In contrast, not having a latrine at home displayed a lower rate for hospital admission (MCBIR 0.68, 95%CI 0.53–0.89, *P* < 0.001). We did not observed any association between malnutrition and mortality with household sanitation facilities after controlling for confounders (Fig. 2 and Additional file 3).

### Herd protection of neighbors’ water and sanitation conditions for morbidity and mortality

#### Water source herd protection

**Fig. 3:**
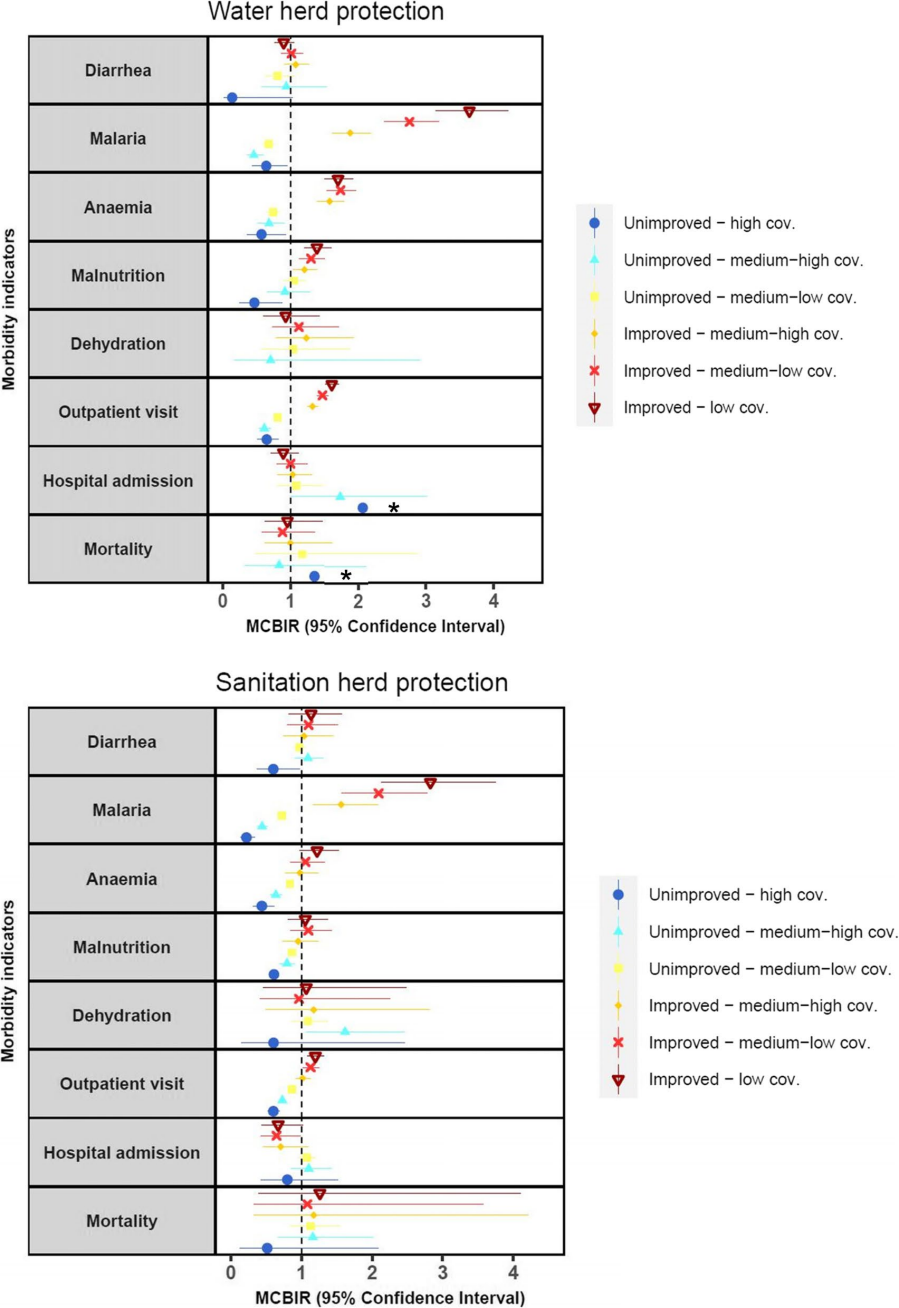
Minimum children-based incidence rates (MCBIR) for diarrhea, malaria, anemia, malnutrition, dehydration, outpatient visit, hospital admission and mortality per household water and sanitation facility used considering neighbors improved water and sanitation conditions coverage during 2012–2015 in Manhiça district adjusted for age, sex, SES, season and distance to health center. The reference categories were “household improved facilities surrounded with high coverage” and “household unimproved facilities surrounded by low coverage”

Children living in a household with an unimproved water facility surrounded by neighbors with high improved water coverage showed a lower rate for malaria, anemia, malnutrition and outpatient visit compared to those living in a household with an unimproved water facility surrounded by neighbors with low improved water coverage. In fact, those surrounded by neighbors with at least medium - low coverage showed a lower rate for malaria, anemia and outpatient visit compared to those surrounded by low coverage. Children living in a household with an improved water facility surrounded by low improved water coverage tripled malaria risk (MCBIR 3.64, 95%CI 3.15–4.21, *P* < 0.001). On the other side, living with improved water conditions but having neighbors with less than high improved water coverage had a higher rate for malaria, anemia, malnutrition and outpatient visit. Diarrhea, dehydration, hospital admission and mortality was not associated with neighbors water coverage considering own household facilities. (Fig. 3 and Additional file 4).

#### Sanitation herd protection

Children living in a household with an unimproved sanitation facility surrounded by high sanitation coverage exhibited a lower rate for diarrhea, malaria, anemia, malnutrition and outpatient visit compared to those surrounded by neighbors with low coverage. Malaria rate was lower by 78% when a child lived in a household with unimproved sanitation conditions surrounded by neighbors with high coverage, and by 56% with medium-high coverage. In addition, malaria rate was three times greater in children living with improved sanitation conditions but surrounded by neighbors with low coverage (MCBIR 2.83, 95%CI 2.13–3.75, *P* < 0.001), twice by medium-low coverage (MCBIR 2.09, 95%CI 1.57–2.78, *P* < 0.001) and 50 % by medium-high coverage (MCBIR 1.56, 95%CI 1.17–2.09, *P* < 0.001) compared to surrounded by high coverage. Moreover, households with improved sanitation surrounded by low coverage and medium-low coverage also displayed a higher outpatient visit rate (MCBIR 1.13, 95%CI 1.02–1.25, *P* < 0.001, MCBIR 1.20, 95%CI 1.08–1.32, *P* = 0.025, respectively). Regarding dehydration, those children that were living in a household with unimproved sanitation conditions and they were surrounded by neighbors with medium-high coverage of sanitation were associated with a higher dehydration rate (MCBIR 1.62, 95%CI 1.06–2.46, *P* < 0.001). Hospital admission and mortality did not show association with sanitation neighbors’ coverage considering own household facilities (Fig. 3 and Additional file 4).

## Discussion

Our analysis showed that water and sanitation facilities used in the household and by household neighbors can affect children’s health. Thus, both should be considered when assessing WASH interventions impact on human health.

Resembling other sub-Saharan regions, the proportion of inhabitants with household improved water facilities progressed each year, while with improved sanitation facilities remained stable during the study period [4]. In our study area, this dynamic could be attributed to local interventions largely focused on water.

Children living in a household using unimproved water and sanitation facilities showed a higher outpatient visit incidence, a human health proxy. Indeed, they showed a greater rate of diarrhea, malaria and anemia. Moreover, neighbors water and sanitation herd protection was observed for outpatient visit and, in particular, for malaria, anemia and malnutrition. Nonetheless, severe morbidity (hospital admission) was associated with household water and sanitation use but not with neighbors improved water and sanitation coverage.

Diarrhea incidence was higher in children living in a household with unimproved water and sanitation facilities. Our diarrhea data collection method is more accurate than self-reporting surveillance [9, 10, 21–24]. Health care-based diarrhea incidence can bias towards severe cases but it is less biased than self-reporting; self-reporting can be affected by recall period and governance claims [25]. Thus, some studies using self-reported data could not find association between diarrhea and water and sanitation although its biological plausibility [9, 10, 21, 23]. In our analysis, surface water doubled diarrhea risk in children. Surface water is affected by rainfalls, which flush enteric pathogens from unimproved latrines or open defecation areas [26, 27]. Thus, we expected that improved sanitation neighbors’ coverage would protect from pathogen infection. In this study, we only observed herd protection when improved sanitation coverage was high, lower improved sanitation neighbor coverage and water neighbor coverage were not associated with diarrhea. Household facilities use might have a stronger influence on diarrhea than neighbor facilities. Indeed, using an unimproved latrine at home showed a greater risk of diarrhea, [22, 28, 29] but open defecation had no association. Certainly, although sanitation infrastructure reduce environmental contamination, latrine dirtiness or poor excreta management augments user’s pathogen exposure compared to open defecation [21].

Malaria and water and sanitation association was evaluated in very few studies. Three studies found no association [29–31] but we observed that household and neighbor’s use of unimproved water and sanitation facilities displayed a higher malaria rate in children. This occurs because vectors might breed and flight from uncovered water storage, surface water or stagnant water around infrastructure [30]. Piped water and improved latrines with a lid could prevent that. Thus, WASH interventions might contribute on malaria control as well.

A superior anemia’s rate associated with household and neighbors using unimproved water and sanitation facilities is supported by other studies [7, 31, 32]. Nevertheless, two cluster-randomized trials observed no association between an intervention on sanitation improvement and anemia. Authors suggest that the reason for that could be lack of participants adherence to their intervention, since adopting behavior change is challenging [9, 33]. Fortunately, this could not occur in our research since we evaluated existent infrastructure but not an intervention.

A higher rate of malnutrition associated with unimproved water facilities was sustained by other studies [8, 34–36]. Regarding sanitation, neighboring sanitation infrastructure was associated with a greater malnutrition rate, while household did not. This is consistent not only with cluster-randomized trials, which suffer from intervention acceptability, but with cross-sectional studies [7, 21–23] and Fuller et al. (2016) model. This model suggested that surrounding households with improved sanitation protects more from stunting than own household facilities [8]. In our study area, pathogen transmission networks causing malnutrition seem more relevant inter-household than intra-household as well.

Dehydration was associated with household and neighbors water facility used. The use of an improved latrine in the household or the use of an unimproved sanitation facility surrounded by neighbors with medium-high coverage were associated with a higher rate of dehydration. Limited research found association between water and sanitation facilities with dehydration. Two studies observed that water provision enhanced schoolchildren fluid intake and hydration [37, 38]. Thus, our restriction to infrastructure exposure but not water quantity might have limited or biased our results.

Mortality did not exhibit any association with household and neighbors’ water and sanitation. The low number of mortality events in our study area could have limited our analysis too. Although mortality was not associated with water and sanitation in this region, others found improved facilities protected it [39–44].

To summarize study limitations, to base our exposure on main water and sanitation infrastructure used could be the main cause to bias our results. Other household practices were not considered (e.g. occasional use of rivers or open defecation), as well as access to, cleanness or operability of infrastructures. Nevertheless, another methodological limitation that has not been mentioned above is the edge effect bias as a result of Kernel density estimation boundaries. Other spatial methodologies could be assessed to evaluate herd protection.

## Conclusions

This study design had the advantage of being a cohort using standardized water and sanitation explanatory variables and clinically determined morbidity outcomes measured objectively. Our herd protection evaluation contributed on driving future research and heightening water and sanitation strategies to improve health. Although the mechanism for herd protection may vary by setting and pathogen transmission cycle, to assess the community-wide protection may improve cost-effectiveness of WASH interventions [32, 45, 46]. Hence, considering the overall water and sanitation impact on health could raise stakeholders’ investment on WASH and enhance WASH related diseases control.

## Supplementary Information

**Additional file 1.** Assets considered for wealth index construction in Manhiça district and its contribution.

**Additional file 2.** Neighbours improved water (A) and sanitation (B) coverage per household during 2012–2015 in Manhiça district.

**Additional file 3.** Minimum children-based incidence rates (MCBIR) for diarrhea, malaria, anaemia, malnutrition, dehydration, outpatient visits, hospital admission and mortality per main water source and sanitation facilities used during 2012–2015 in Manhiça district adjusted for age, sex, socioeconomical index score, season and distance to health post.

**Additional file 4.** Minimum children-based incidence rates (MCBIR) for diarrhoea, malaria, anaemia, malnutrition, dehydration, outpatient visits, hospital admission and mortality per main water source and sanitation facility used in the household considering neighbours water and sanitation improved conditions coverage during 2012–2015 in Manhiça district adjusted for age, sex, socioeconomical index score, season and distance to health post.

## Data Availability

The datasets generated and/or analysed during the current study are not publicly available due to ethical and legal reasons but are available from the corresponding author on reasonable request.

## Abbreviations

CI: Confidence interval
CISM: Centro de Investigação em Saúde de Manhiça
DSS: Demographic Surveillance System
MCA: Multiple Correspondence Analysis
MCBIR: minimum children-based incidence rate
SDG: Sustainable Development Goal
SES: Socioeconomic status
WASH: Water, Sanitation and Hygiene
