# Supplementary material for: Neighbors’ use of water and sanitation facilities can affect children’s health: a cohort study in Mozambique using a spatial approach
Source: BMC Public Health. 2022 May 16;22:983. doi: 10.1186/s12889-022-13373-9 (PMC9109333; doi:10.1186/s12889-022-13373-9)
Supplement: Supplementary file 1 — Additional file 1. Assets considered for wealth index construction in Manhiça district and its contribution. [file 12889_2022_13373_MOESM1_ESM.docx]

**Supplementary Table S1. Assets considered for wealth index construction in Manhiça district and its contribution.**

| **Asset** | **Category** | **Overall inertia (over 1)** | **Contribution to the 1st dimension (over 1)** | **Coordinate of dimension 1 (score 1)** |
| --- | --- | --- | --- | --- |
| House construction type | Chalet | 0.023 | 0.022 | 0.855 |
|  | Apartment | 0.000 | 0.000 | 1,582 |
|  | Palhota* | 0.013 | 0.002 | -1,842 |
|  | Reed hut | 0.025 | 0.023 | -1,022 |
|  | Wood/Zinc hut | 0.006 | 0.004 | -1,442 |
|  | Other | 0.000 | 0.000 | 0.278 |
| Construction material of the main division | All made of reed and straw covered | 0.013 | 0.003 | -1,635 |
|  | All made of reed and zinc covered | 0.027 | 0.026 | -0.958 |
|  | Mud plaster and straw covered | 0.000 | 0.000 | -0.707 |
|  | Mud plaster and zinc plates covered | 0.000 | 0.000 | -0.420 |
|  | All wood/zinc | 0.001 | 0.000 | -0.656 |
|  | Brick/blocks and other precarious | 0.012 | 0.004 | 0.523 |
|  | All brick/blocks | 0.035 | 0.040 | 1,868 |
|  | Other | 0.001 | 0.000 | 0.812 |
| Kitchen location | Inside | 0.041 | 0.040 | 4,155 |
|  | Outside | 0.002 | 0.002 | -0.179 |
| Kitchen coverage | Yes | 0.011 | 0.011 | 0.683 |
|  | No | 0.008 | 0.008 | -0.508 |
| Main cooking fuel | Firewood | 0.009 | 0.010 | -0.469 |
|  | Coal | 0.028 | 0.030 | 1,984 |
|  | Gas | 0.017 | 0.014 | 5,060 |
|  | Electricity | 0.017 | 0.015 | 4,183 |
|  | Fusel | 0.000 | 0.000 | 0.954 |
|  | Other | 0.000 | 0.000 | 1,121 |
| Electricity supply | Yes | 0.059 | 0.064 | 1,726 |
|  | No | 0.037 | 0.041 | -1,094 |
| Telephone | Yes | 0.002 | 0.002 | 0.203 |
|  | No | 0.013 | 0.014 | -1,438 |
| Radio | Yes | 0.016 | 0.019 | 0.886 |
|  | No | 0.012 | 0.014 | -0.670 |
| Video/dvd | Yes | 0.062 | 0.065 | 1,744 |
|  | No | 0.039 | 0.041 | -1,104 |
| Fridge | Yes | 0.069 | 0.079 | 2,469 |
|  | No | 0.021 | 0.024 | -0.751 |
| Car or tractor | Yes | 0.023 | 0.026 | 2,964 |
|  | No | 0.001 | 0.002 | -0.169 |
| Television | Yes | 0.063 | 0.067 | 1,801 |
|  | No | 0.038 | 0.040 | -1,078 |
| Computer | Yes | 0.032 | 0.032 | 4,103 |
|  | No | 0.001 | 0.001 | -0.146 |
| Stove | Yes | 0.053 | 0.061 | 3,158 |
|  | No | 0.007 | 0.008 | -0.393 |
| Farming | Yes | 0.003 | 0.003 | -0.261 |
|  | No | 0.014 | 0.012 | 1,034 |
| Literacy | Yes | 0.010 | 0.010 | 0.516 |
|  | No | 0.021 | 0.021 | -1,069 |
| Head of the household education | Without | 0.011 | 0.011 | -0.533 |
|  | Primary | 0.015 | 0.013 | 1,010 |
|  | Secondary | 0.020 | 0.018 | 3,136 |
|  | High | 0.015 | 0.011 | 5,241 |
|  | Not applicable | 0.000 | 0.000 | -0.240 |
|  | Other | 0.001 | 0.000 | 0.681 |
| Head of the household occupation | Primary sector | 0.002 | 0.001 | 0.324 |
|  | Industry, construction, mining | 0.002 | 0.001 | 0.519 |
|  | Education, health | 0.021 | 0.020 | 2,883 |
|  | Tourism, commerce | 0.001 | 0.001 | 1,047 |
|  | Defence, protection or security | 0.001 | 0.001 | 0.826 |
|  | Informal employment/self-employment | 0.003 | 0.002 | 0.776 |
|  | Unemployed, retired, pensioner | 0.013 | 0.013 | -0.805 |
|  | Incapacitated | 0.000 | 0.000 | -0.475 |
|  | Others | 0.003 | 0.002 | 0.434 |
|  | Student | 0.005 | 0.004 | -0.886 |

*Palhota: Moçambican rounded dwelling of construction with regional available materials.
