# Supplementary material for: Neighbors’ use of water and sanitation facilities can affect children’s health: a cohort study in Mozambique using a spatial approach
Source: BMC Public Health. 2022 May 16;22:983. doi: 10.1186/s12889-022-13373-9 (PMC9109333; doi:10.1186/s12889-022-13373-9)

**Supplementary Figure S2. Neighbours improved water (A) and sanitation (B) coverage per household during 2012-2015 in Manhiça district.**

**A B**


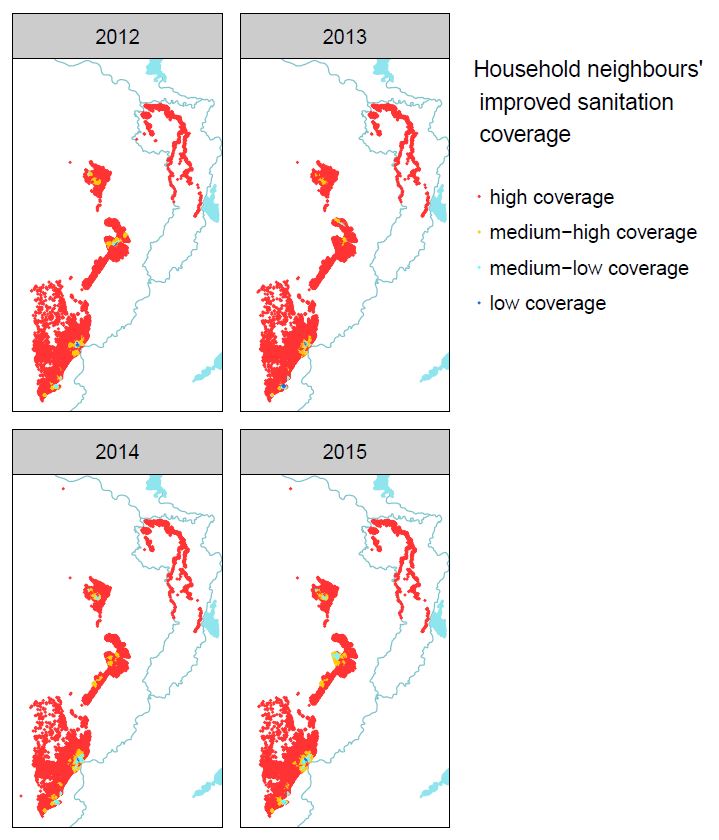

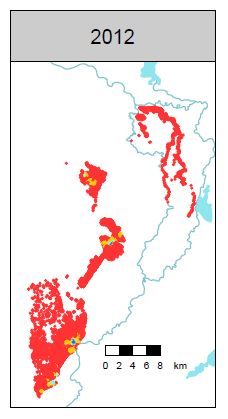

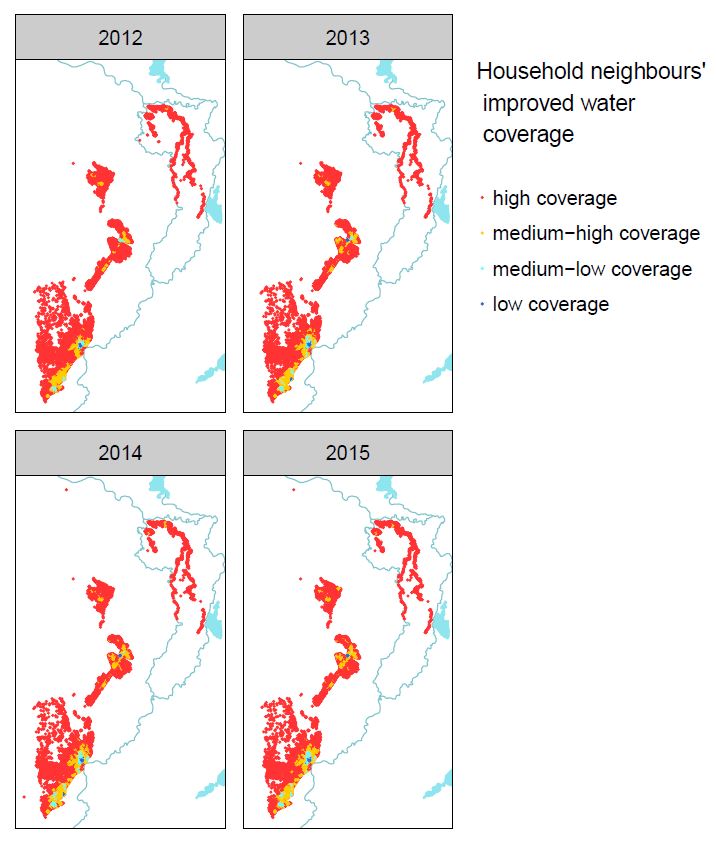


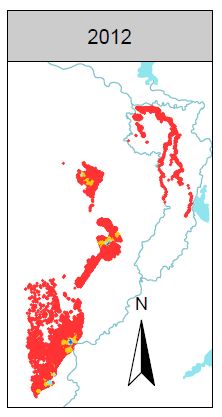

Supplement: Supplementary file 2 — Additional file 2. Neighbours improved water (A) and sanitation (B) coverage per household during 2012–2015 in Manhiça district. [file 12889_2022_13373_MOESM2_ESM.docx]
