# Supplementary material for: Neighbors’ use of water and sanitation facilities can affect children’s health: a cohort study in Mozambique using a spatial approach
Source: BMC Public Health. 2022 May 16;22:983. doi: 10.1186/s12889-022-13373-9 (PMC9109333; doi:10.1186/s12889-022-13373-9)
Supplement: Supplementary file 3 — Additional file 3. Minimum children-based incidence rates (MCBIR) for diarrhea, malaria, anaemia, malnutrition, dehydration, outpatient visits, hospital admission and mortality per main water source and sanitation facilities used during 2012–2015 in Manhiça district adjusted for age, sex, socioeconomical index score, season and distance to health post. [file 12889_2022_13373_MOESM3_ESM.docx]

**Supplementary Table S3. Minimum children-based incidence rates (MCBIR) for diarrhea, malaria, anaemia, malnutrition, dehydration, outpatient visits, hospital admission and mortality per main water source and sanitation facilities used during 2012-2015 in Manhiça district adjusted for age, sex, socioeconomical index score, season and distance to health post.**
