# Supplementary material for: Neighbors’ use of water and sanitation facilities can affect children’s health: a cohort study in Mozambique using a spatial approach
Source: BMC Public Health. 2022 May 16;22:983. doi: 10.1186/s12889-022-13373-9 (PMC9109333; doi:10.1186/s12889-022-13373-9)
Supplement: Supplementary file 4 — Additional file 4. Minimum children-based incidence rates (MCBIR) for diarrhoea, malaria, anaemia, malnutrition, dehydration, outpatient visits, hospital admission and mortality per main water source and sanitation facility used in the household considering neighbours water and sanitation improved conditions coverage during 2012–2015 in Manhiça district adjusted for age, sex, socioeconomical index score, season and distance to health post. [file 12889_2022_13373_MOESM4_ESM.docx]

**Supplementary Table S4. Minimum children-based incidence rates (MCBIR) for diarrhoea, malaria, anaemia, malnutrition, dehydration, outpatient visits, hospital admission and mortality per main water source and sanitation facility used in the household considering neighbours water and sanitation improved conditions coverage during 2012-2015 in Manhiça district adjusted for age, sex, socioeconomical index score, season and distance to health post.**

MCBIR for children living in a household with unimproved water conditions surrounded by neighbors with improved water high coverage could not be obtained, the number of participants with those conditions was low.
